# The Relationship Between Dietary Intake and Sleep Quality in Endurance Athletes

**DOI:** 10.3389/fspor.2022.810402

**Published:** 2022-03-03

**Authors:** Kamiah Moss, Yan Zhang, Andreas Kreutzer, Austin J. Graybeal, Ryan R. Porter, Robyn Braun-Trocchio, Meena Shah

**Affiliations:** ^1^Department of Kinesiology, Texas Christian University, Fort Worth, TX, United States; ^2^Harris College of Nursing and Health Sciences, Texas Christian University, Fort Worth, TX, United States; ^3^School of Kinesiology and Nutrition, College of Education and Human Sciences, University of Southern Mississippi, Hattiesburg, MS, United States

**Keywords:** dietary intake, sleep quality, endurance athlete, chronotype, sleep behavior

## Abstract

Many endurance athletes have poor sleep quality which may affect performance and health. It is unclear how dietary intake affects sleep quality among athletes. We examined if sleep quality in endurance athletes is associated with consumption of fruit, vegetables, whole grains, dairy milk, and caffeinated beverages. Two hundred thirty-four endurance athletes (39.5 ± 14.1 year) participated in a survey. Participants provided information on demographics, anthropometry, sleep behavior and quality, and dietary intake *via* questionnaires. Sleep quality was assessed using the Athlete Sleep Screening Questionnaire (ASSQ) with a global score (ASSQ-global) and subscales including sleep difficulty (ASSQ-SD), chronotype (ASSQ-C), and disordered breathing while sleeping (ASSQ-SDB). A general linear model (GLM), adjusted for age, body mass index, sleep discomfort, sleep behavior, gender, race, and ethnicity, showed that higher caffeinated beverage intake was related to poorer global sleep quality (*p* = 0.01) and increased risk for disordered breathing while sleeping (*p* = 0.03). Higher whole grain intake was associated with a morning chronotype and lower risk for sleep issues (*p* = 0.01). The GLM did not reveal a relationship between sleep quality and dairy milk, fruit, and vegetable intake. In conclusion, caffeinated beverages and whole grain intake may influence sleep quality. This relationship needs to be confirmed by further research.

## Introduction

Sleep inadequacy, characterized as having insufficient sleep duration (<7 h), decreased subjective sleep satisfaction, increased sleep latency or time to fall asleep, unrestful sleep, day-time sleepiness, and/or feeling fatigued during the day, is common among athletes with 50–78% suffering from one or more of these issues (Samuels, 2008; Swinbourne et al., 2016; Walsh et al., 2021). Inadequate sleep among athletes has been found to negatively affect physical performance (Halson, 2014), impair immune responses (i.e., increased susceptibility to illness) (Walsh, 2018), and increase risk for injury (Milewski et al., 2014). Inadequate sleep also lowers psychomotor performance such as attention, concentration, speed processing and decisive tasks in athletes (Monleon et al., 2018). These variables are vital for optimal performance in this population. In addition to psychomotor problems, inadequate sleep causes or modulates pain and increases physical and psychological stress in this group (Halson, 2014). Both pain and stress have been found to negatively affect athletic performance (Otter et al., 2015; Flood et al., 2017).

Possible causes for poor sleep quality and/or quantity among athletes include the low priority of sleep relative to training demands, lack of awareness of the role of sleep in performance, and scheduling constraints (Simpson et al., 2017). Because of the high susceptibility for poor sleep quality among athletes and the negative effects of sleep inadequacy on performance and health, exploring strategies that could improve sleep outcomes among athletes is warranted. More specifically, there has been increased interest in recent years in the role of dietary intake on sleep quality. Possible dietary factors related to sleep quality may be intake of fruit, vegetables, dairy milk, whole grains, and caffeine.

According to a recent systematic review of studies in the general population, most but not all studies have found a protective association of healthy foods such as fruit, vegetables, whole grain, and dairy milk, and sleep quality, and many have shown a detrimental relationship between caffeine consumption and sleep health (Godos et al., 2021). Moreover, consumption of a Mediterranean type of diet (high in plant based foods) may have a protective role on sleep quality (Godos et al., 2019). Higher fruit or vegetable consumption has been linked to longer sleep duration (Imaki et al., 2002; Stamatakis and Brownson, 2008; Noorwali et al., 2018), fewer sleep disturbances (Zuraikat et al., 2020a), better sleep quality (Zuraikat et al., 2020a), and improved efficiency or total time asleep relative to time in bed (Zuraikat et al., 2020a). Studies on specific fruit found that kiwifruit and tart cherry juice increase sleep duration and efficiency (Lin et al., 2011; Howatson et al., 2012). Kiwifruit also reduces sleep latency and awakenings (Lin et al., 2011), and tart cherry juice increases sleep satisfaction (Nødtvedt et al., 2017). Lower whole grain consumption is associated with less sleep (Haghighatdoost et al., 2012; Jansen et al., 2020), more sleep latency (Zuraikat et al., 2020b), and impaired sleep quality (Nisar et al., 2019). Further, individuals that have less whole grain, vegetable, and fruit intake have more of an evening chronotype, i.e., they prefer to conduct activities in the evening which increases risk for sleep issues (Kanerva et al., 2012; Patterson et al., 2016). Moreover, milk and dairy foods has been linked to better sleep quality possibly because dairy foods contain the amino acid tryptophan a precursor for melatonin (Komada et al., 2020).

There are no studies to our knowledge that have assessed the role of fruit, vegetable, and whole grain intake on sleep among endurance athletes. However, several randomized studies have evaluated the effect of dairy milk and caffeine intake on sleep quality among athletes. Consuming dairy milk at breakfast decreased sleep latency among Japanese soccer athletes (Kawada et al., 2016). More frequent dairy milk consumption decreased the risk for poor sleep quality among female but not male Japanese athletes (Yasuda et al., 2019). As with dairy milk, a number of randomized controlled studies have assessed the role of caffeine intake on sleep outcomes among athletes. Higher caffeine consumption caused increased trouble sleeping (e.g., difficulty falling asleep and increased awakenings during the night) (Pallarés et al., 2013), sleep latency (Ali et al., 2015; Dunican et al., 2018), restlessness (Ali et al., 2015), wakefulness (Ali et al., 2015), likelihood for insomnia (Salinero et al., 2014), decreased sleep quality (Ali et al., 2015; Ramos-Campo et al., 2019) and sleep efficiency (Dunican et al., 2018) among athletes.

A major limitation of the current literature is that the relationship between fruit, vegetable, and whole grain intake and sleep quality has not been examined among athletes despite the fact that significant sleep issues have been reported in this population (Imaki et al., 2002; Stamatakis and Brownson, 2008; Haghighatdoost et al., 2012; Patterson et al., 2016; Noorwali et al., 2018; Nisar et al., 2019; Jansen et al., 2020; Zuraikat et al., 2020a,b). It is important to examine this relationship given that poor sleep quality is linked to lower psychomotor (Monleon et al., 2018) and physical performance (Bulbulian et al., 1996), increased risk for injury (Johnston et al., 2020), and impaired health (Tonder et al., 2016) among endurance athletes. Moreover, participation in endurance sports is rapidly increasing (Jeukendrup, 2011) further underscoring the significance of examining potential dietary factors that may mitigate the risk for poor sleep quality. Therefore, the purpose of this study was to examine if sleep quality in endurance athletes such as cyclists, runners, and triathletes is related to the consumption of fruit, vegetables, whole grains, dairy milk, and caffeinated beverages. Based on previous research, we hypothesized that participants who consume higher quantities of fruit, vegetables, whole grains, or dairy milk will have better sleep quality, while athletes who consume larger amounts of caffeinated beverages will have poorer sleep quality.

## Materials and Methods

### Recruitment and Participants

Endurance athletes were recruited *via* email and social media groups using a digital flyer. They were also recruited by sending the flyer to online organizations that channel communications between endurance athletes and their teams and coaches, and by word of mouth.

Two hundred thirty-four participants (95.2% from North America and 4.8% from elsewhere), who self-identified as an endurance athlete (i.e., engaging in sports that are fueled by aerobic energy production which can last for hours to days) (Vernillo et al., 2016; Savoldelli et al., 2017) based on competitive sport or training style, were included in the study. Individuals under the age of 18 years, those who did not self-identify as an endurance athlete and compete in training activities for endurance-based sports were excluded. The Institutional Review Board approved the study and each participant read and signed an approved informed consent document. Data was collected during the COVID-19 pandemic from June 2020 to February 2021.

### Measurements

#### Demographics

All anthropometric and demographic measures were obtained *via* a self-reported questionnaire. Height was reported in inches and weight was reported in pounds. Body mass index (BMI) was calculated by dividing body weight (kg) by height (meter) squared. BMI was used to categorize participants as underweight (<18.5 kg/m^2^), normal weight (18.5–24.9 kg/m^2^), overweight (25.0–29.9 kg/m^2^), or obese (≥30.0 kg/m^2^).

Demographic information collected included age, sex (male and female), ethnicity (Hispanic and non-Hispanic), race (White, African American, Asian, American Indian, Native Hawaiian, multiracial, and other), education (high school diploma or lower, vocational training, some college, college degree, and graduate degree or higher), and household income (< $20,000, $20,000–34,999, $35,000–49,999, $50,000–99,999, and >$100,000 USD).

#### Type of Sport

Self-reported information on the type of endurance sport and competitive level was collected *via* questionnaire. The endurance sport categories included cycling, running, triathlon, and other (e.g., para-cycling, race walking, rowing, swimming, and wheelchair racing).

#### Sleep Behavior

Sleep behavior was assessed using the Athlete Sleep Behavior Questionnaire (ASBQ), an 18-item validated questionnaire (Cronbach's alpha = 0.63) designed to identify sleep behaviors among athletes (Driller et al., 2018). The ASBQ measures maladaptive sleep behaviors and is utilized to provide recommendations on sleep hygiene in athletic populations (Driller et al., 2018). The ASBQ asks athletes questions such as taking prolonged naps (≥ 2 h) during the day, training in the evening, getting up in the night to use the restroom, worrying, using light emitting devices, drinking alcohol (<4 h) before bedtime, room temperature in the bedroom, and bed and pillow comfort (Driller et al., 2018). Participants answered the ASBQ questions by selecting the 5-point Likert scale choices including never, rarely, sometimes, frequently, or always. ASBQ global scores were calculated by adding up each item, and a higher score indicates poorer sleep behavior. An ASBQ global score of ≤ 36 indicates good, 37-41 neither good nor poor, and ≥42 poor sleep behavior (Driller et al., 2018).

#### Sleep Difficulty Due to Discomfort

The participants provided a self-reported response to a question on whether or not they have had trouble sleeping due to muscle pain, numbness, aching, soreness, or twitching in the survey.

#### Sleep Quality

Sleep quality was assessed using the Athlete Sleep Screening Questionnaire (ASSQ). The ASSQ is a 16-item validated screening tool (Cronbach's alpha = 0.74) to assess sleep quality over the last month among athletic populations (Samuels et al., 2016; Bender et al., 2018). The ASSQ provides validated cut off points to detect clinically significant sleep difficulties among athletes. A higher score indicates poorer sleep quality (Samuels et al., 2016; Bender et al., 2018). The ASSQ subscales include sleep difficulty (SD), chronotype (C), and sleep disordered breathing (SDB) (Bender et al., 2018). ASSQ-SD was categorized as having none (0–4), mild (5–7), moderate (8–10), or severe (11–17) SD (Bender et al., 2018). ASSQ-C was categorized as morning (>4) or evening (≤ 4) type (Bender et al., 2018). Chronotype indicates the time of day that an individual prefers to conduct their daily activities (Mazri et al., 2020). Those with an evening chronotype are at increased risk for poor sleep quality (Mazri et al., 2020). ASSQ-SDB was categorized as difficulty breathing (≥1) or no difficulty breathing (<1) during sleep (Bender et al., 2018). ASSQ-SDB indicates if the participant reported either snoring loudly or had sleep apnea (choking, gasping, and/or stop breathing while sleeping) or both (Bender et al., 2018).

#### Dietary Intake

Information on usual intake of fruit (fresh, frozen, canned, or dried), vegetables (raw, cooked, canned, or frozen; excluding white potatoes), dairy milk (whole, 2, 1, $\frac{1}{2}$%, or non-fat skim), whole grains (bread, tortillas, oatmeal, cereal, rice, pasta, or popcorn), and caffeinated beverages (coffee, tea, soda, energy drinks) was collected over the last month. Fruit quantities were categorized as consuming <1, 1–2, 3–4, 5–6, 7–8 and > 8 servings/d, vegetable as <1, 1–2, 3–4, 5–6, 7–8 and >8 servings/d, whole grain quantities were categorized by consuming <1, 1–2, 3–4, 5–6, 7–8, 9–10,11–12, >12 servings/d, dairy milk as <1, 1–2, 3–4, 5–6, 7–8 and >8 cups/d, and caffeinated beverages as <1, 1–1.5, >1.5–2, >2–2.5 and >2.5 cups/d. Serving sizes for each food group assessed were defined according to standards set by the Dietary Guidelines for Americans (USDA USDHHS U&U, 2020).

### Procedures

Eligible subjects provided information on demographics, anthropometry, dietary intake, and sleep habits *via* questionnaires on Qualtrics using their own computer, cell phone, or another electronic device. The Qualtrics link was available to the participants in the recruitment flyer. Participants completed the questionnaires once based on their usual dietary intake and sleep habits over the last month.

### Statistical Analysis

After a preliminary assessment of the sample, the demographic and dietary variable categories with <5% of the sample distribution were regrouped within each variable, and the final categories are shown in the results and tables.

Participant characteristics are presented as percentages. Sleep quality (ASSQ global score) by participant characteristics is presented as mean ± standard deviation (SD). The bivariate relationships between sleep quality (ASSQ global scores) and participant characteristics were assessed using a one-way ANOVA for characteristics with more than two categories (age, education, household income, type of sport, BMI, and sleep behavior) and an independent samples t-test for variables with two categories (race, ethnicity, gender, and sleep discomfort).

The bivariate relationships between sleep quality (ASSQ global scores) and participants' fruit, vegetable, whole grain, dairy milk, and caffeinated beverage intakes were assessed using a one-way ANOVA. The same test was also used to examine the relationship between each ASSQ subscale (SD, C, and SDB) and dietary intake. Follow-up analyses were conducted to identify significant differences in ASSQ global and subscale scores by dietary categories.

A general linear model (GLM) was performed to further examine the multivariate relationship between the intake of fruit, vegetables, whole grains, dairy milk, and caffeinated beverages and sleep quality (ASSQ global and subscales scores), while controlling for age, BMI, sleep discomfort, sleep behavior, gender, race, and ethnicity. Age, BMI, and sleep behavior were included as covariates (continuous variables) and gender, race, sleep discomfort, and ethnicity were included as fixed factors (dichotomous variables) in the GLM.

Data were analyzed using IBM SPSS version 26 (Armonk, NY, USA).

## Results

### Demographic Characteristics

Mean (SD) age was 39.5 ± 14.1 years. Participant characteristics for the total sample are presented in Table 1. Slightly more than 50% of the participants were 18–39 year, female, and had a household income of ≥$100,000. More than 80% of the participants were white, non-Hispanic, and had a college or graduate degree. More participants identified themselves as runners (37.6%) and triathletes (33.8%) than cyclists (20.5%). About one-third of the participants were overweight or obese, had poor sleep behavior, and felt discomfort while sleeping due to pain, numbness, aching, soreness, or twitching.

**Table 1:** Sleep quality (ASSQ global score) by participant characteristics.

**Participant characteristics**	**Total sample**	**ASSQ global**
	**(*n* = 234)**	**(*n* = 234)**
	***n* (%)**	
**Age (year)**		
18–39	121 (51.7)	19.8 ± 4.13
40–59	88 (37.6)	22.0 ± 4.27^a^^**^
≥60	24 (10.3)	22.1 ± 4.50^a^^*^
NR	1 (0.4)	
**Gender**		
Male	104 (44.4)	21.0 ± 4.49
Female	121 (51.7)	20.6 ± 4.30
NR	9 (3.8)	
**Race**		
White	208 (88.9)	21.0 ± 4.36
Non-White	23 (9.8)	20.0 ± 4.21
NR	3 (1.3)	
**Ethnicity**		
Hispanic or Latino	23 (9.8)	20.3 ± 4.18
Non-Hispanic or Latino	196 (83.8)	21.0 ± 4.47
NR	15 (6.4)	
**Education**		
High school diploma/vocational training or lower	11 (4.7)	19.6 ± 4.88
Some college	33 (14.1)	20.4 ± 4.76
College degree	75 (32.1)	20.5 ± 4.31
Graduate degree or higher	115 (49.1)	21.3 ± 4.26
NR	0 (0.0)	
**Household income**		
< $20,000 USD	13 (5.6)	19.8 ± 3.79
$20,000–$49,999 USD	19 (8.1)	20.8 ± 5.52
$50,000–$74,999 USD	27 (11.5)	21.3 ± 4.94
$75,000–$99,999 USD	23 (9.8)	20.2 ± 4.49
≥$100,000 USD	124 (53.0)	21.2 ± 3.98
NR	28 (12.0)	
**Endurance sport**		
Cycling	48 (20.5)	21.9 ± 4.48
Running	88 (37.6)	20.9 ± 4.06
Triathlon	79 (33.8)	19.9 ± 4.52
Other	19 (8.1)	21.9 ± 4.37
NR	0 (0.0)	
**Body mass index (kg/m** ^**2**^ **)**		
Underweight	8 (3.4)	19.8 ± 2.77
Normal	139 (59.4)	20.7 ± 4.48
Overweight	44 (18.8)	20.8 ± 4.47
Obese	28 (12.0)	21.7 ± 4.19
Height and/or weight NR	15 (6.4)	
**Athlete Sleep Behavior global score**		
Good sleep behavior	74 (31.6)	19.6 ± 3.93^b^^**^
Neither good nor poor sleep behavior	73 (31.2)	20.5 ± 4.45
Poor sleep behavior	83 (35.5)	22.1 ± 4.33
NR	4 (1.7)	
**Discomfort during sleep due to (pain, numbness, aching, soreness, or twitching)**		
Yes	81 (34.6)	21.8 ± 4.15
No	153 (65.4)	20.3 ± 4.41^c^^**^
NR	0 (0.0)	

*ASSQ, Athlete Sleep Screening Questionnaire; NR, not reported; BMI, body mass index*.

*ASSQ, global scores are presented as mean ± standard deviation*.

*One-way analysis of variance was used to compare the relationship between sleep quality (ASSQ global score) and age, education, income, endurance sport type, BMI categories and sleep behavior*.

*Independent t-test was used compare sleep quality (ASSQ global score) by gender, race, ethnicity, and sleep discomfort*.

a *Compared to 18–39 years*.

b *Compared to poor sleep behavior*.

c *Compared to yes*.

* *p < 0.05,*

** *p < 0.01*.

### ASSQ Global Scores by Participant Characteristics

ASSQ global scores (sleep quality) were significantly different by age groups [*F*_(2, 228)_ = 8.36, *p* < 0.001, partial η^2^ = 0.068], sleep behavior levels [*F*_(2, 225)_ = 7.06, *p* = 0.001, partial η^2^ = 0.059] (Table 1), and sleep discomfort [*t*_(172.59)_ = 2.62, *p* = 0.01, *r* = 0.20]. Participants who were 18–39 years old had better sleep quality (lower scores) compared to those who were 40–59 years (*p* = 0.01) and ≥60 years (*p* = 0.04) old. Participants with good sleep behavior had significantly better sleep quality (lower scores) vs. those with poor sleep behavior (*p* = 0.01). Presence of sleep discomfort due to pain, numbness, aching, soreness, or twitching was related to poorer sleep quality (higher scores) compared to no sleep discomfort. There were no significant differences in sleep quality (ASSQ global scores) by gender, race, ethnicity, education, household income, type of sport, or BMI categories.

### Bivariate Analysis of Sleep Quality (ASSQ Global) and Subscale Scores by Dietary Intake

Bivariate relationships between dietary intake and ASSQ global and subscale scores are presented in Table 2. There was a significant relationship between ASSQ global scores (sleep quality) and caffeinated beverage [*F*_(4, 189)_ = 8.87, *p* < 0.001, partial η^2^ = 0.158] but not fruit, vegetable, dairy milk, or whole grain consumption. *Post hoc* tests revealed that those who consumed 1.5 cups/d or less of caffeinated beverages had significantly better sleep quality (lower scores) compared to those who consumed more caffeinated beverages (*p* < 0.05).

**Table 2:** Sleep quality (ASSQ global) and subscale scores by dietary intake.

**Dietary intake**	**Total sample** **(*n* = 234) *n* (%)**	**ASSQ global** **(*n* = 234)**	**ASSQ-SD** **(*n* = 234)**	**ASSQ-C** **(*n* = 234)**	**ASSQ-SDB** **(*n* = 234)**
**Fruits**					
<1 serving/d	42 (17.9)	20.1 ± 4.10	6.02 ± 2.82	8.67 ± 2.74	0.50 ± 0.77
1–2 servings/d	112 (47.9)	21.0 ± 4.01	6.05 ± 3.01	9.64 ± 2.70	0.30 ± 0.64
3-4 servings/d	61 (26.1)	20.8 ± 4.97	6.08 ± 3.65	9.33 ± 2.82	0.11 ± 0.41^a^^*^
≥ 5 servings/d	15 (6.4)	20.9 ± 5.02	6.47 ± 3.07	9.73 ± 2.74	0.40 ± 0.74
Not reported	4 (1.7)				
**Vegetables**					
<1 serving/d	17 (7.3)	21.0 ± 4.91	7.06 ± 3.27	8.88 ± 1.97	0.18 ± 0.53
1–2 servings/d	98 (41.9)	20.4 ± 4.27	5.84 ± 2.93	9.26 ± 2.63	0.32 ± 0.67
3–4 servings/d	81 (34.6)	21.2 ± 4.26	6.27 ± 3.33	9.53 ± 2.98	0.28 ± 0.64
≥5 servings/d	36 (15.4)	20.8 ± 4.75	5.86 ± 3.32	9.75 ± 2.75	0.25 ± 0.50
Not reported	2 (0.9)				
**Whole grains**					
<1 serving/d	25 (10.7)	20.4 ± 4.99	6.96 ± 3.59	8.24 ± 2.88	0.44 ± 0.77
1–2 servings/d	115 (49.1)	20.9 ± 4.02	6.13 ± 2.89	9.26 ± 2.93	0.37 ± 0.68
3–4 servings/d	68 (29.1)	20.8 ± 4.56	5.56 ± 3.13	10.2 ± 2.28^b^^*^	0.10 ± 0.39^b^^*^
≥5 servings/d	25 (10.7)	20.8 ± 5.06	6.60 ± 3.91	9.00 ± 2.29	0.28 ± 0.68
Not reported	1 (0.4)				
**Dairy milk**					
<1 cup/d	170 (72.6)	20.9 ± 4.35	6.15 ± 3.16	9.45 ± 2.71	0.28 ± 0.62^c^^*^
1–2 cups/d	46 (19.7)	20.1 ± 4.39	5.87 ± 3.12	8.98 ± 3.00	0.17 ± 0.49^c^^*^
≥3 cups/d	17 (7.3)	22.0 ± 4.64	6.29 ± 3.53	9.82 ± 2.16	0.71 ± 0.92
Not reported	1 (0.4)				
**Caffeinated beverages**					
<1 cup/d	20 (8.5)	18.1 ± 2.75	4.75 ± 2.61^f^^*^	9.05 ± 2.28	0.20 ± 0.52
1–1.5 cups/d	58 (24.8)	19.2 ± 3.78	5.38 ± 2.77^f^ ^*^	9.31 ± 2.49	0.10 ± 0.41
>1.5–2 cups/d	48 (20.5)	21.6 ± 3.96^d^^*^, ^e^ ^*^	5.98 ± 2.85	10.1 ± 2.40	0.40 ± 0.74
>2–2.5 cup/d	36 (15.4)	22.5 ± 4.64^d^^*^, ^e^ ^*^	7.19 ± 3.58	9.06 ± 2.84	0.44 ± 0.77
>2.5 cups/d	32 (13.7)	23.0 ± 4.56^d^^*^, ^e^^*^	6.78 ± 2.93	9.09 ± 3.34	0.47 ± 0.72
Not reported	40 (17.1)				

*ASSQ, Athlete Sleep Screening Questionnaire; ASSQ-SD, Athlete Sleep Screening Questionnaire - sleep difficulty; ASSQ-C, Athlete Sleep Screening Questionnaire - chronotype; ASSQ-SBD, Athlete Sleep Screening Questionnaire - sleep disordered breathing*.

*Data are presented as mean ± standard deviation unless otherwise specified*.

*One-way analysis was used to analyze the relationship between global sleep scores and sleep subscales and fruit, vegetable, whole grain, dairy milk, and caffeinated beverage intake*.

a *Compared to < 1 serving/d of fruit*.

b *Compared to < 1 serving/d of whole grains*.

c *Compared to ≥3 cups/d of dairy milk*.

d *Compared to < 1 of cup/d of caffeinated beverages*.

e *Compared to 1–1.5 cups/d of caffeinated beverages*.

f *Compared to >2–2.5 cups/d of caffeinated beverages*.

* *p < 0.05*.

There was a significant relationship between ASSQ-SD (sleep difficulty) and caffeinated beverage consumption [*F*_(4, 189)_ = 3.54, *p* = 0.01, partial η^2^ = 0.070]. Participants who consumed 1.5 or fewer cups/d of caffeinated beverages had significantly lower sleep difficulty compared to those who consumed >2–2.5 cups/d (*p* < 0.05). Fruit, vegetable, whole grain, or dairy milk consumption was not related to sleep difficulty.

There was a significant relationship between ASSQ-C (chronotype) and whole grain consumption [*F*_(3, 229)_ = 3.67, *p* = 0.02, partial η^2^ = 0.046] but not fruit, vegetable, dairy milk, or caffeinated beverage intake. Participants who consumed <1 serving/d of whole grains had lower ASSQ-C scores, associated with a more evening chronotype, compared to those who consumed 3–4 servings/d (*p* = 0.03).

There was a significant relationship between ASSQ-SDB (sleep disordered breathing) and fruit [*F*_(3, 226)_ = 3.36, *p* = 0.02, partial η^2^ = 0.043], whole grain [*F*_(3, 229)_ = 3.24, *p* = 0.02, partial η^2^ = 0.041], dairy milk [*F*_(2, 230)_ = 4.63, *p* = 0.01, partial η^2^ = 0.039], and caffeinated beverage [*F*_(4, 189)_ = 2.78, *p* = 0.03, partial η^2^ = 0.056] but not vegetable intake. Those who consumed 3–4 servings/d of fruits had lower scores, indicating less difficulty breathing while sleeping, compared to those who consumed <1 serving/d (*p* = 0.03). Participants who consumed <1 servings/d of whole grains had higher scores than those who consumed 3–4 servings/d (*p* = 0.04). Those who consumed ≥ 3 cups/d of dairy milk had higher scores compared to those consumed less milk (*p* < 0.05). Although there was an overall significant relationship between caffeinated beverage intake and ASSQ-SDB, there were no individual differences among the groups.

### GLM: Relationship Between ASSQ Global and Subscale Scores and Dietary Intake

The GLM assessing the relationship between dietary predictors (caffeinated beverages, dairy milk, whole grain, fruit, and vegetables) and ASSQ global and subscales (ASSQ-SD, ASSQ-C, and ASSQ-SDB), controlling for age, gender, race, ethnicity, BMI, sleep behavior, and sleep discomfort, showed a significant positive relationship between sleep quality (ASSQ global scores) and caffeinated beverage consumption [*F*_(4, 139)_ = 3.60, *p* = 0.01, partial η^2^ = 0.094] presented in Table 3. As caffeinated beverage consumption increased, ASSQ global scores increased, indicating worse sleep quality. There was also a significant positive relationship between ASSQ-SDB and caffeinated beverage intake [*F*_(4, 139)_ = 2.73, *p* = 0.03, partial η^2^ = 0.073]. As caffeinated beverage intake increased, ASSQ-SDB increased, indicating increased difficulty breathing while sleeping. There was a significant positive relationship between ASSQ-C and whole grain consumption [*F*_(3, 139)_ = 3.88, *p* = 0.01, partial η^2^ = 0.077]. Higher whole grain intake was associated with increased ASSQ-C scores (more of a morning chronotype), indicating decreased risk for sleep issues. The remaining dietary variables were not related to sleep quality in the GLM analysis.

**Table 3:** GLM: relationship between ASSQ Global and subscale scores and dietary intake.

**Predictor**	**ASSQ Global^a^**			**ASSQ-SD^b^**			**ASSQ-C^c^**			**ASSQ-SDB^d^**		
	**SS**	**df**	**F**	**SS**	**df**	**F**	**SS**	**df**	**F**	**SS**	**df**	**F**
Fruits	23.20	3	0.504	7.46	3	0.329	4.54	3	0.247	1.02	3	1.05
Vegetables	20.94	3	0.455	16.68	3	0.736	2.57	3	0.140	2.02	3	2.09
Whole Grains	28.53	3	0.619	19.30	3	0.852	71.36	3	3.88^**^	1.78	3	1.84
Dairy Milk	10.70	2	0.348	21.04	2	1.39	11.06	2	0.902	0.568	2	0.879
Caffeinated Beverages	220.98	4	3.60^**^	20.16	4	0.667	16.33	4	0.666	3.53	4	2.73^*^
Residual	2,135.14	139		1,049.98	139		851.93	139		44.88	139	

*SS, sum of squares; df, degrees of freedom*.

a *ASSQ Global R squared = 0.294 (Adjusted R squared = 0.177)*.

b *ASSQ-SD R squared = 0.255 (Adjusted R squared =0.131)*.

c *ASSQ-C R squared = 0.271 (Adjusted R squared = 0.150)*.

d *ASSQ-SDB R squared = 0.254 (Adjusted R squared = 0.130)*.

*GLM was used to analyze the relationship between global sleep scores and sleep subscales and fruit, vegetable, whole grain, dairy milk, and caffeinated beverage intake controlling for age, gender, race, ethnicity, BMI, sleep behavior and sleep discomfort*.

* *p < 0.05,*

** *p < 0.01*.

## Discussion

This is the first study that examined how consumption of fruit, vegetables, and whole grains affect sleep quality in endurance athletes, and found that lower whole grain intake is related to increased evening sleep chronotype. The present study also showed that increased caffeinated beverage intake is associated with poor sleep quality and increased disordered breathing while sleeping.

Consuming > 1.5 cups of caffeinated beverages per day was related to lower sleep quality than consuming a lower amount. This would be equal to > 150 mg of caffeine in brewed coffee. Salinero et al. (2014) reported higher rates of insomnia in endurance athletes when they were given an energy drink containing 3 mg of caffeine per kg body weight vs. placebo prior to exercise. Based on their average weight, male and female subjects in the study by Salinero et al. (2014) received 228 and 189 mg of caffeine, respectively. The results from this study as well as our study suggest that limiting coffee to 1.5 cups per day may be prudent in athletes with sleep inadequacy.

Lower whole grain consumption was linked to a better chronotype score in both the bivariate and GLM analyses. Bivariate analysis also showed that lower whole grain intake was related to a higher score for disordered breathing. Based on the ASSQ questionnaire, the prevalence of disordered breathing was 19.7% in our participants. Our results on whole grain consumption and sleep quality are corroborated by several cross-sectional studies among non-athletes. Reid et al. (2019) reported that obstructive sleep apnea, a type of disordered breathing, was associated with lower intake of whole grains in a multi-ethnic population from the U.S. Kanerva et al. (2012) found that adults in Finland who consumed fewer whole grains had a chronotype toward eveningness. Lower whole grain intake was also associated with shorter sleep duration and more sleep latency (Zuraikat et al., 2020b) in women, and less sleep in the general US population (Jansen et al., 2020). Moreover, sleep quality was found to be higher among medical students in Pakistan who consumed more whole grains (Nisar et al., 2019). The results on whole grain intake and sleep quality need to be confirmed by laboratory-controlled polysomnography studies that specifically manipulate whole grain intake while controlling for all other variables potentially influencing sleep.

Higher dairy milk intake was related to increased disordered breathing while sleeping but only in the bivariate analysis where the other predictors were not controlled for. Our results are not consistent with the previous literature. For instance, Kawada et al. (2016) reported that consuming dairy milk for 20 days vs. no dairy milk resulted in decreased sleep latency among male soccer athletes. Further, Yasuda et al. (2019) found that consuming dairy milk ≤ 2 vs. 3 or more days/week was associated with increased risk for poor sleep quality in female but not male Japanese elite Olympic athletes. The inconsistent results between our study and the previous studies may be due to the fact that there was not much variability in dairy milk consumption in the present study with 72.6% consuming less than 1 serving of dairy milk. Moreover, the previous studies did not control for intake of whole grains and caffeine which we found to be related to sleep in the GLM analysis.

Bivariate analysis showed that lower fruit consumption was associated with increased disordered breathing while sleeping in the present study. Stamatakis and Brownson (2008) and Noorwali et al. (2018) reported shorter sleep duration among Japanese factory workers and British women who consumed less fruit. Moreover, Zuraikat et al. (2020a) reported fewer sleep disturbances among American women who ate more fruit.

The mechanisms by which dietary intake influences sleep quality is not fully understood. An important circadian hormone which regulates sleep is melatonin (Pereira et al., 2020). Melatonin production occurs in the pineal gland at night which results in sleepiness (Halson, 2014; Pereira et al., 2020). One of the proposed mechanisms is that caffeine consumption during the day reduces the production of melatonin leading to sleep disruptions at night (Shilo et al., 2002). Consumption of carbohydrate rich foods such as whole grains may promote sleep by increasing tryptophan which is a precursor for serotonin (Halson, 2014; Pereira et al., 2020). Serotonin is converted into melatonin (Jiki et al., 2018; Pereira et al., 2020). Additionally, butyric acid made when dietary fiber from whole grains is fermented by bacteria in the gut and polyphenols (plant based chemicals) in plant foods such as grains and fruit may lead to increased production of a neurotransmitter, gamma-aminobutyric acid (GABA) (Halson, 2014; Nisar et al., 2019; Noorwali et al., 2019). GABA inhibits neural activity in the central nervous system resulting in decreased sleep latency and increased sleep duration (Halson, 2014; Noorwali et al., 2019). Retinoic acid (vitamin A) synthesized from beta-carotene in fruit has been shown to regulate the circadian sleep cycle, sleep stages, and sleep duration (Navigatore-fonzo et al., 2014; Ji et al., 2017). Further, studies have shown that diets rich in grains, fruit, and vegetables increase gut microbial diversity (Van Der Merwe, 2021) which has been positively correlated with increased sleep efficiency and duration (Smith et al., 2019).

Our study is limited by the cross-sectional design in which causation may not be inferred. Also, there may be reverse causality, i.e., inadequate sleep quality may lead to poor dietary intake. Another limitation is that the study was conducted using a convenience sample which may limit the generalizability of the results. The data was self-reported, and the participants may have reported their dietary intake and/or sleep behavior to be better than what it is. The data on dietary intake was collected over the last month, and this may not have captured seasonal variations in food consumption. Cycling or running on a hilly vs. a flat terrain may lead to higher energy expenditure (Vernillo et al., 2016; Savoldelli et al., 2017). In our sample, 81.3% of cyclists, 76.1% of runners, and 94.9% of triathletes were road cyclists, road runners, and road triathletes, respectively. Global sleep scores were not different by type of cyclists, runners, or triathletes. We did not limit the study to a specific level of endurance athlete. Majority (80.8%) of our athletes identified themselves as recreational athletes, and there was no difference in sleep quality by level of athlete when controlling for age. In addition, sleep quality was not assessed using objective measures such as polysomnography or actigraphy. Further, the majority of the participants were non-Hispanic white which decreases the ability to generalize the sample to all endurance athletes. We had a wide age range which affected sleep quality. We addressed this by adjusting for age in the GLM analysis on diet and sleep quality. Our sample was not large enough to run the analysis by age sub-groups. The study results may have been affected by the COVID-19 pandemic. Trabelsi et al. (2021) reported that the COVID-19 lockdown was associated with lower sleep quality and total physical activity energy expenditure in adults >55 years. Many (10.3%) of our participants were older adults. We did not assess water intake of the participants which could affect their sleep quality. Sleep disturbances have been reported by Chamari et al. (2016) in athletes who avoid any food or fluid from dawn to sunset during Ramadan. The average BMI of our sample was 23.4 ± 3.4 and 22.4 ± 2.8 kg/m^2^ in male and female athletes, respectively, and is lower than that shown among runners in the Running USA report (Running USA., 2013). Nevertheless, the prevalence of overweight or obesity in our sample may be overestimated because BMI does not distinguish lean mass from fat mass. Witt and Bush (2005) have reported that many athletes are misclassified as overweight based on BMI classification. Assessment of body fat is needed to classify athletes as normal or overweight.

The study has several strengths. To our knowledge, this is the first study to examine the relationship between sleep quality and fruit, vegetable, and whole grain intake in endurance athletes. The study utilized validated questionnaires which were designed to assess sleep quality and behavior in athletes. The sleep issues in our participants were similar to that among athletes in previous studies. We found that 30.7% of athletes had clinically significant moderate or severe sleep difficulty. These results are consistent with a clinical validation study in which 25.1% of athletes were reported to have clinically significant sleep difficulty (Bender et al., 2018). The majority of our participants had a morning chronotype and low prevalence of disordered breathing which is similar to that reported by other studies among athletes (Quan et al., 2007; Lastella et al., 2016).

To infer causation, randomized controlled studies examining the effect of a healthy diet rich in plant foods vs. a standard diet on sleep quality in athletes are needed. These studies should also assess melatonin levels, psychomotor and physical performance since melatonin affects sleep which in turn affects performance. Moreover, melatonin levels are influenced by diet as noted earlier, and melatonin supplementation in sleep deprived collegiate student athletes improves psychomotor and physical performance (Paryab et al., 2021).

In summary, the present study revealed that increased caffeinated beverage intake and decreased intake of whole grains were associated with poor sleep quality in endurance athletes. The findings of this study suggest that dietary intake may influence sleep quality. To improve sleep quality among athletes, dieticians and coaches should promote more whole grains and restrict caffeinated beverage consumption especially close to bedtime, in addition to encouraging healthy sleep behaviors. Randomized controlled studies examining the effect of more nourishing diets on sleep outcomes are needed. Moreover, sleep outcomes need to be assessed using both subjective and objective instruments.

## Data Availability Statement

The raw data supporting the conclusions of this article will be made available by the authors, without undue reservation.

## Ethics Statement

The studies involving human participants were reviewed and approved by Texas Christian University Institutional Review Board. The patients/participants provided their written informed consent to participate in this study.

## Author Contributions

KM: conceptualization, writing—original draft preparation, project administration, methodology, formal analysis, and investigation. YZ: methodology, formal analysis, investigation, and writing—review and editing. AK and AG: conceptualization, project administration, investigation, and writing—review and editing. RP: investigation and writing—review and editing. RB-T: conceptualization and writing—review and editing. MS: conceptualization, methodology, writing—review and editing, supervision, and funding acquisition. All authors contributed to the article and approved the submitted version.

## Funding

This study was supported in part by the Harris College of Nursing and Health Sciences Graduate Student Research Grant.

## Conflict of Interest

The authors declare that the research was conducted in the absence of any commercial or financial relationships that could be construed as a potential conflict of interest.

## Publisher's Note

All claims expressed in this article are solely those of the authors and do not necessarily represent those of their affiliated organizations, or those of the publisher, the editors and the reviewers. Any product that may be evaluated in this article, or claim that may be made by its manufacturer, is not guaranteed or endorsed by the publisher.
